# COVID-19 pandemic and adolescent mental health in China: Micro evidence and socioeconomic mechanisms

**DOI:** 10.3389/fpsyg.2022.1041376

**Published:** 2022-11-14

**Authors:** Boou Chen, Chunkai Zhao, Xing Li, Jin Liu

**Affiliations:** ^1^School of Economics, Nanjing University of Finance and Economics, Nanjing, China; ^2^College of Economics and Management, South China Agricultural University, Guangzhou, China; ^3^College of Economics and Management, Southwest University, Chongqing, China; ^4^Institute of Finance and Economics Research, Shanghai University of Finance and Economics, Shanghai, China; ^5^Institute for Urban-Rural Development, Shanghai University of Finance and Economics, Shanghai, China

**Keywords:** COVID-19 pandemic, adolescents, mental health, socioeconomic mechanisms, Difference-in-Differences model, China

## Abstract

Although the impact of the COVID-19 pandemic on adolescent mental health has received sufficient attention in the medical and public health fields, analysis from the social science perspective is still lacking. By regarding the shock of COVID-19 pandemic as a quasi-natural experiment, this study adopts the Difference-in-Differences (DID) model and large microdata from Shandong Province, China, to identify the causal effect of the COVID-19 pandemic on the mental health of senior high school students. We find that the COVID-19 pandemic results in an increase of 2.5677 points in adolescent psychological problem scores, equivalent to an average decrease of 29.93% in mental health. Furthermore, results of mechanism tests show that this negative impact of the COVID-19 pandemic on adolescent mental health can be explained by a reduction in social trust, as well as widening inequalities caused by the digital divide and family income gap. Moreover, the estimates suggest that the COVID-19 pandemic has a greater negative influence on the mental health of boys and urban adolescents. Our study complements the research field on the impact of the COVID-19 pandemic on adolescent mental health and the potential socioeconomic mechanisms from a new perspective. These findings provide insights into how to safeguard adolescent mental health in China and other countries in the pandemic prevention and post-pandemic era.

## Introduction

The COVID-19 pandemic has led to more than 605 million infections and nearly 6.5 million deaths worldwide. As one of the major global public health crises, the impact of the COVID-19 pandemic on people’s physical and mental health has received widespread attention from a large number of scholars. In addition to the direct effect of the COVID-19 pandemic on the mental health of patients (Pfefferbaum and North, 2020; Vindegaard and Benros, 2020; Kumar and Nayar, 2021), it may also indirectly impact people’s mental health through multiple channels. On the one hand, the economic recession and unemployment caused by the COVID-19 pandemic may undermine people’s mental health (Van Dorn et al., 2020; Ginsburgh et al., 2021; Wildman, 2021). On the other hand, during the pandemic prevention and control period, the risk perceptions of public health crises, the spread of negative information, family isolation measures and the maintenance of social distance may also bring negative emotions such as anxiety and worry (Chen et al., 2020; Commodari et al., 2020; Ding et al., 2020; Huang and Zhao, 2020; Zhou et al., 2020; Tian et al., 2022).

For immature adolescents, the COVID-19 breakout is also making a profound difference in their learning, lives and health. Not surprisingly, the impact of the COVID-19 pandemic on adolescent mental health has received ample attention in the medical and public health fields (e.g., Racine et al., 2020; Xiang et al., 2020; Hu and Qian, 2021; Shek et al., 2021; Wright et al., 2021; Kim et al., 2022). Most studies have observed a trend of deterioration in the overall mental health of adolescents following the COVID-19 pandemic and a significant increase in the number of reported symptoms of depression and anxiety (Adibelli and Sümen, 2020; Shek et al., 2021; Wu et al., 2021; Cao et al., 2022). For example, Duan et al. (2020) surveyed 3,613 Chinese students through online recruitment and found that 22.28% of them experienced anxiety and depression during the COVID-19 pandemic. Liang et al. (2020) also revealed that nearly 40.4% adolescents in the sample were found to be prone to psychological problems and 14.4% the sampled youth with Post-traumatic stress disorder (PTSD) symptoms after the occurrence of COVID-19. Worse, for some adolescents with prior mental illness or chronic disease, they experienced higher levels of psychological distress, depression, and behavioral problems (Jefsen et al., 2021). Some studies even found that the COVID-19 pandemic led to an increased incidence of suicidal ideation, suicide, and non-suicidal self-harm in adolescents (Banati et al., 2020; Durante and Lau, 2022).

China is one of the countries in the world with the most stringent prevention and control of the COVID-19 pandemic. Similarly, existing studies, through some local or online survey data, supported the conclusion that the COVID-19 pandemic worsened Chinese adolescent mental health (e.g., Chen et al., 2020; Duan et al., 2020; Qi et al., 2020; Zhang et al., 2020; Chi et al., 2021; Qu et al., 2021; Cao et al., 2022). First, in the context of COVID-19 prevention and control, the epidemic prevention department requires to keep social distance, reduce social interaction, and shorten the time of outdoor interaction, which is detrimental to the mental health of adolescents (Duan et al., 2020; Li Y. et al., 2021; Samji et al., 2022; Tang et al., 2020). Second, the epidemic has caused most schools to move traditional classes online, increasing students’ access to electronic devices, which has a negative impact on their physical and mental health (Dong et al., 2020; Hu and Qian, 2021). Third, for some families without electronic devices or with low income, the inequality brought by the epidemic may further hurt the mental health of adolescents (Hu and Qian, 2021; Cheshmehzangi et al., 2022; Samji et al., 2022).

Although the potential impact of the COVID-19 pandemic on adolescent mental health has attracted considerable scholarly interest, these studies have mainly stayed in the medical and public health fields. Few studies explored the effect of the COVID-19 pandemic on adolescent mental health from social science perspectives (Banati et al., 2020; Brodeur et al., 2021; Jaspal and Breakwell, 2022). More importantly, little is still known about the potential socioeconomic mechanisms of the COVID-19 pandemic breaking adolescent mental health. In addition, most of these studies in China relied on small-scale questionnaires or online surveys (e.g., Chen et al., 2020; Duan et al., 2020; Cao et al., 2022), lacking the support of large-scale micro-data. Therefore, unlike these previous studies, we use a large micro-database, the Database of Youth Health (DYH), which consisted of a multi-wave survey conducted annually in the academic year 2015/2016, 2016/2017, 2017/2018, and 2020/2021, and contained nearly 100,000 sampled students from 186 middle schools in 17 cities of Shandong Province, China (Zhang et al., 2022). This database is conducted by Shandong University and National Population Health Data Center in China, and contains a large number of variables on students’ mental health, basic information, and socio-economic status, providing the feasibility of our study (Yi et al., 2019; Dong et al., 2020, 2021). Moreover, the shock of the COVID-19 pandemic provides a perfect quasi-natural experiment, which creates conditions for us to use the Difference-in-Differences (DID) model, a widely used approach in the field of social sciences (e.g., O’Connell et al., 2022; Zhao et al., 2022; Zhao and Yan, 2022), to identify the impact of the COVID-19 pandemic on adolescent mental health in China. In terms of the measurement of mental health, we used the Strengths and Difficulties Questionnaire (SDQ), which is widely used in research related to adolescent mental health (e.g., Goodman et al., 2000; Rotheray et al., 2014; Kersten et al., 2017; Hu and Qian, 2021; Ravens-Sieberer et al., 2022), and measured adolescents’ mental health in five dimensions: Emotional symptoms, Conduct problems, Inattention, Peer relationship problems, and Prosocial problems.

The contributions of this study are reflected in the following three aspects. First, our study enriches the literature on the impact of the COVID-19 pandemic on adolescent mental health. In particular, using an identification method commonly used in the social sciences, we confirm the causal relationship between the COVID-19 pandemic and adolescent mental health loss in China (Adibelli and Sümen, 2020; Banati et al., 2020; Xiao et al., 2020). Furthermore, because of the importance of mental health as a component of human capital (Goldsmith et al., 1997; Graff and Neidell, 2013), our findings suggest a negative impact of the COVID-19 pandemic on human capital (O’Connell et al., 2022).

Second, this study further supplements the potential socioeconomic mechanisms that the COVID-19 pandemic affects adolescent mental health. Previous studies mainly analyzed the negative impact of the COVID-19 pandemic on adolescent mental health from three aspects: society, family, and media (Duan et al., 2020; Evans et al., 2020; Qu et al., 2021; Rogers et al., 2021; Shek et al., 2021; Samji et al., 2022). In this paper, we innovatively increase the investigation on channels in terms of social trust, digital divide, and family income gap. In particular, previous research rarely analyzed the negative impact of the COVID-19 pandemic on mental health in terms of economic factors (Wildman, 2021).

Third, in terms of theoretical contributions, from the perspective of adolescent mental health loss, our research implies that the shock of the COVID-19 pandemic may exacerbate social inequalities. Specifically, we analyze the possible mechanistic roles of social trust, digital divide and family income gap from the theoretical level. Thus, by adopting the case from China, this study further supplements to rapidly growing literature that focused on social inequalities caused by critical public health incidents, like the COVID-19 pandemic (Van Dorn et al., 2020; Fisher and Ryan, 2021; Ginsburgh et al., 2021; Wildman, 2021). Additionally, our findings provide insights into how to develop more rational public policies to enhance the welfare and utility of these vulnerable groups during the pandemic and post-pandemic era.

## Theoretical mechanisms and research hypotheses

**Figure 1:**
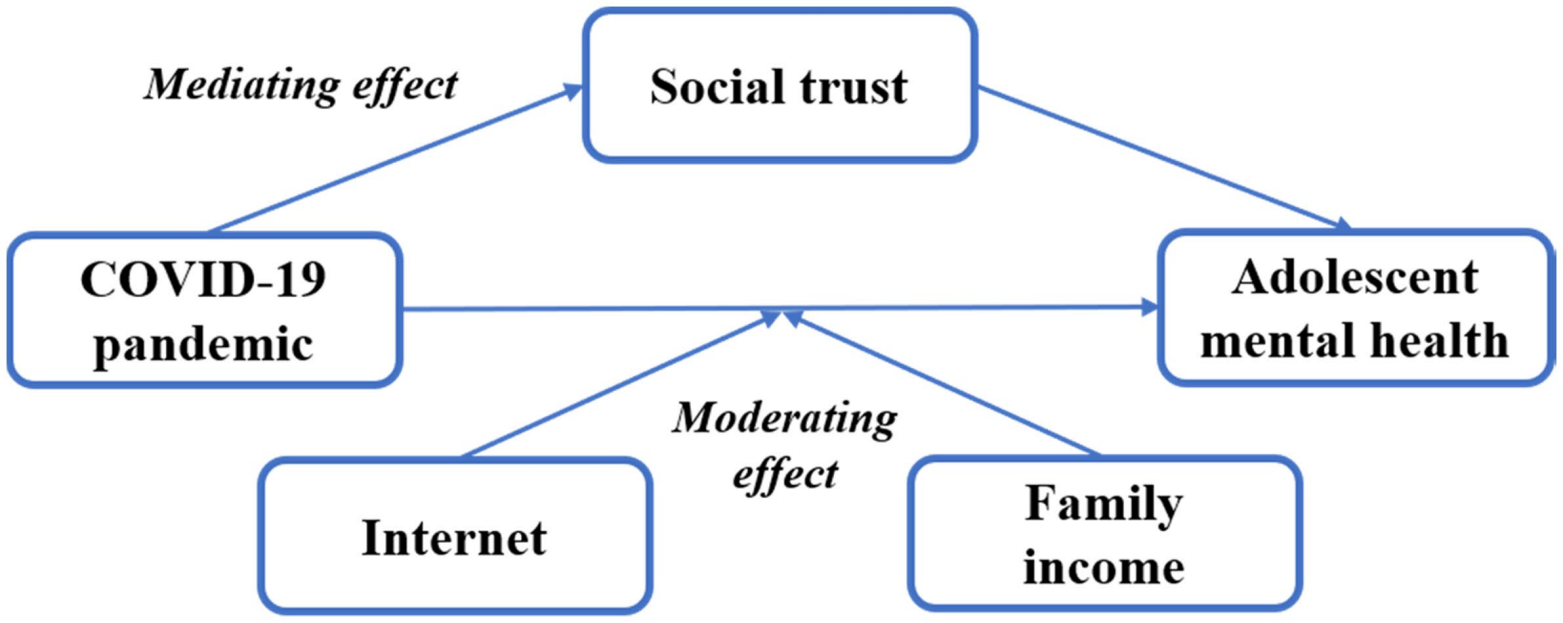
The mechanisms of the COVID-19 pandemic on adolescent mental health.

In this section, based on a socioeconomic perspective, we theoretically analyzed the mechanisms through which the COVID-19 pandemic affects adolescent mental health from both subjective adolescent factors (psychological and behavioral factors) and objective family factors. More specifically, based on the data available in the questionnaire, we focused on the mediating effect of adolescent social trust in the above-mentioned effects at the level of subjective factors. In terms of objective family factors, we explored the moderating effects of digital divide (family Internet accessibility) and family income, and also discussed the association between the digital divide, family income gap and adolescent mental health during the COVID-19 pandemic. The details of mechanisms are shown in Figure 1.

### Social trust

Negative public health events such as epidemics often pose a huge challenge to social trust (Kye and Hwang, 2020; Woelfert and Kunst, 2020; Aassve et al., 2021). When the epidemic breaks out, people instinctively become fearful of the risks, illnesses, and deaths caused by epidemics (Jefsen et al., 2021; Kumar and Nayar, 2021; Li W. et al., 2021), reducing social interactions and communications. Meanwhile, it is important to note that individual risk perceptions of the COVID-19 pandemic may also have a differential impact on social trust and related social behaviors, which includes distance perception, affective risk perception and cognitive risk perception, and thus on the mental health (Commodari et al., 2020; Ding et al., 2020).

Additionally, the inconvenience associated with epidemic prevention and control may allow people to transmit negative emotions about the virus or disease to patients and grassroots organizations (Kye and Hwang, 2020; Woelfert and Kunst, 2020), resulting in a social phenomenon of “generalized stigmatization” (Lin et al., 2022), which further exacerbates attitudes of social mistrust. Worse, during the epidemic, a series of factors, such as value conflicts and resource competitions, also combine to exacerbate the transfer and spread of this mistrust (Siegrist et al., 2000). Furthermore, epidemics are often accompanied by the spread of various rumors, making a higher opportunity cost of acquiring true information. In theory, adopting heuristic decision-making strategies or rules of thumb becomes the optimal choice (Nunn and Wantchekon, 2011). In the situation of the outbreak and spread of COVID-19, the costs for individuals to know if others are carrying the virus can be very high, as rigorous medical testing is required to determine (Kye and Hwang, 2020). Therefore, as a low-cost optimal strategy, people are more willing to choose to trust their acquaintances based on a rule of thumb (Brodeur et al., 2021). In contrast, for strangers, the level of trust naturally decreases due to the excessive transaction costs of information acquisition and screening.

Adolescents are in a vulnerable position both in terms of access to authentic information and screening of information. With the advice of their parents, schools, and local governments, they tend to comply with the COVID-19 pandemic prevention and control measures more strictly (Dong et al., 2020; Qu et al., 2021). Thus, adolescents’ outdoor activities and communications are further reduced, which may decrease their social trust (Qi et al., 2020; Samji et al., 2022). Even in public, they are also likely to be more highly worried and fearful, especially in crowded places or by public transportation, and have significantly less social trust in strangers and deliberately stay away from them (Kye and Hwang, 2020; Woelfert and Kunst, 2020; Aassve et al., 2021). More importantly, adolescents are still immature and are more likely to take relatively conservative measures to resist risk in the face of negative events such as the COVID-19 pandemic (Zhao and Li, 2022). In cases where information on the illness of others is difficult to obtain, they tend to lower their social trust to avoid exposing themselves to risky events (Aassve et al., 2021).

In terms of the situation in China, as the Chinese government has implemented strict COVID-19 pandemic control measures, the itineraries of infected individuals are published, which makes it less difficult for people to obtain information about this disease (Xiao et al., 2020; Cao et al., 2022). Despite this, there are still strict requirements for vaccinations, nucleic acid testing, masks and social distancing, making adolescents aware that the risk of infection still exists (Shek et al., 2021). At this point, reducing trust in strangers remains a good option. Given that the positive impact of social trust on individual mental health is widely recognized (e.g., Fahmi et al., 2019; Roychowdhury, 2021), we propose our first hypothesis:

*Hypothesis 1*: The COVID-19 pandemic is likely to reduce adolescents’ social trust, especially with strangers in public, which is detrimental to their mental health.

### Digital divide

With the popularity of the Internet, digital tools have played an important role in the prevention and control of the COVID-19 pandemic (Shah et al., 2020). For example, in China, relying on support from the Internet and big data technology, people scan health codes, trip codes, and nucleic acid codes to cooperate with various epidemic prevention and control measures (Guo et al., 2022). In the event of infections, digital technology can accurately identify their activity trajectory and analyze the source of viral infection, contributing to epidemic control and special medical care for patients (Zhu et al., 2022). Nonetheless, the phenomenon of the digital divide in epidemic prevention and control has increasingly drawn the attention of scholars (e.g., Dong et al., 2020; Duan et al., 2020; Qi et al., 2020; Hu and Qian, 2021; Samji et al., 2022). In particular, COVID-19 has led to the need for students to study online. For some students in rural or remote areas, they face a large digital divide in online education.

First, there is a large gap between households in rural areas and urban households in terms of Internet access, Internet speed, and flexibility of electronic equipment and software use, especially in developing countries like China (Li and Ranieri, 2013). Epidemic-induced online teaching and learning may lead to difficulties for rural students to keep up and greatly reduce their learning effectiveness (Samji et al., 2022). Second, at the level of the skills divide, many teachers in rural schools do not have the flexibility to use digital technology to present three-dimensional and diverse knowledge content due to limitations (Warf, 2019). Also, students are also not able to make more innovative connections or expand their horizons (Looker and Thiessen, 2003). Third, in terms of the empowerment gap, online education technology can provide students with a channel for self-empowerment and access to more social capital (Hess and Leal, 2001). However, most rural schools are unable to provide sufficient and excellent online education resources, further widening the urban–rural education gap.

Previous studies confirmed that the digital divide further evolved into inequalities in human capital, increasing rural–urban differences in physical and mental health (e.g., Ennis et al., 2012; Shah et al., 2020). Therefore, we put forward our second hypothesis:

*Hypothesis 2*: The COVID-19 pandemic widens the digital divide, leading to impaired adolescent mental health.

### Family income gap

We consider that the third mechanism by which the COVID-19 pandemic influences adolescent mental health is the family income gap. As one of the “black swan” incidents, the COVID-19 pandemic has hit people’s income in several ways, causing a further widening of the social income gap. On the one hand, the COVID-19 pandemic has resulted in an economic recession, a contraction in foreign trade and international investment, and a reduction in fiscal sources (Crossley et al., 2021; Ginsburgh et al., 2021; Wildman, 2021), which brings about the decrease in the guaranteed social benefits available to socially vulnerable groups, especially for developing countries (Qian and Fan, 2020; Brodeur et al., 2021). On the other hand, the COVID-19 pandemic has caused higher unemployment rates and lower income. Evidence from the United States, Sweden, India, and Korea show that COVID-19 negatively affected the labor force participation rate, working hours, and residents’ income, bringing about a significant increase in unemployment (Aum et al., 2021; Desai et al., 2021; Hensvik et al., 2021). However, wealthy groups are adept at using emergencies such as epidemics to protect their property and even make a profit (Leach et al., 2021), such as entering the related industries for epidemic prevention and control. Also, this income gap may be exacerbated by a series of economic stimulus policies adopted because of the epidemic (Ginsburgh et al., 2021).

There is no doubt that family income is closely related to adolescent mental health. Studies from psychology show that adolescents from families in higher socioeconomic classes have significantly higher levels of positive affect, life satisfaction, and mental health than those in lower economic classes (McLaughlin et al., 2012; Coley et al., 2018). In addition, adolescents from low-income families generate perceptions of their own class economic backwardness and higher levels of low self-esteem and depression through upward social comparisons (Baird et al., 2013). They are more likely to have psychological problems such as obsessive–compulsive symptoms, depression, and psychoticism, impairing their personality development and long-term psychological health (McLaughlin et al., 2012; Reiss, 2013). Therefore, the widening family income gap caused by the COVID-19 pandemic may lead to mental health loss among adolescents from relatively low-income families due to a range of negative issues, such as lack of socioeconomic resources, less emotional care, and inferior position in social comparison (Coley et al., 2018; Hu and Qian, 2021; Lu et al., 2021). More specifically, the outbreak of the COVID-19 pandemic caused a shortage of livelihood resources in the short term. Short-term supply shocks may result in price increases for some essential goods, which are obviously very detrimental to low-income households, while high-income households are less affected. Increased inequality in access to necessities is likely to exacerbate some psychological problems of adolescents in low-income households, such as excessive worries about the future and panic due to the lack of food and medical supplies. Therefore, we develop the third hypothesis:

*Hypothesis 3*: Widening family income gap from the shock of the COVID-19 pandemic further worsens the mental health of adolescents.

It has to be noted that some mechanisms are not independent of each other and it is very difficult to really pin down each mechanism. For example, adolescents from low-income families face a higher probability of counting the digital divide (Ennis et al., 2012). However, there are also different meanings of the moderating effects of the digital divide and the income gap. The different focus of these two mechanisms implies that the COVID-19 pandemic has led to different aspects of social inequality, both of which deserve the attention of government, the public, and academia.

## Data and methods

### Data

The data used in this study is from two databases. First, the data on the COVID-19 pandemic of cities in China comes from the COVID-19 Timely Dynamic Tracking Database released by SINA.^1^ SINA is an online media company serving China and the global Chinese community. According to the official data released by local governments, COVID-19 Timely Dynamic Tracking Database from SINA has continuously collected and released real-time data on COVID-19 in various cities in China since January 20, 2020, mainly including the cumulative (or new) number of confirmed cases, deaths, cured cases, and so on.

Second, the data on students’ mental health and basic characteristics come from the DYH, which is conducted by Shandong University and National Population Health Data Center in China. The DYH program consisted of a multi-wave survey conducted annually in the academic year 2015/2016, 2016/2017, 2017/2018, and 2020/2021, and investigated 99,327 junior or senior high school students from 186 secondary schools in 17 cities of Shandong province in China (Zhang et al., 2022). This database is the first open shared dataset about Chinese adolescents’ health, and contains rich variables about students’ basic information, mental health, social-economic status, social interaction, nutrition and diet, and so forth (Yi et al., 2019; Dong et al., 2020, 2021). More importantly, to our knowledge, the DYH is one of the few large micro databases that can provide information before and after the spread of COVID-19 in China. Therefore, the DYH provide a good data basis for exploring the impact of the COVID-19 pandemic on adolescent mental health.

In our research, considering the adaptability of the data, we used the data from the DYH in the academic year 2017/2018 and 2020/2021 for analysis, and excluded samples with missing values for the explanatory variable, explained variables, and control variables. In addition, it is important to note that since the DYH program only investigated the mental health status of senior high school students, the study ultimately included a sample of 12,096 senior high school students from 10 cities in Shandong Province in China.

### Variables

#### Measures of adolescent mental health

Our study refers to the Strengths and Difficulties Questionnaire (SDQ) to evaluate adolescent mental health, which is a widely used method to capture adolescents’ self-reported mental health (e.g., Goodman et al., 2000; Rotheray et al., 2014; Kersten et al., 2017; Hu and Qian, 2021; Ravens-Sieberer et al., 2022). The SDQ contains 5 subscales: emotional symptoms, conduct problems, hyperactivity/inattention, peer relationship problems, prosocial behavior, and 5 items are set for each subscale.^2^ Each item contains three options: not true (0), somewhat true (1), and certainly true (2), and then the score of each subscale can be obtained by adding up the scores for its 5 constituent items. A higher score indicates more serious psychological problems for the first four subscales, that is, the worse status of mental health. Differently, a higher score indicates the better mental health for the prosocial behavior subscale.

The DYH contains a lot of questions about student mental health, which are also self-reported by students. Therefore, referring to the subscales of the SDQ, we constructed an indicator system to measure adolescent mental health according to the relevant questions and information available in the DYH, as shown in Table A1 in the Appendix. Specifically, we set three relevant items in the DYH for each subscale, and each item contains four options: never (0), not serious (1), moderate (2), relatively serious (3), and very serious (4). The score for each subscale, ranging from 0–12, were calculated by summing the corresponding 3 items. Different from the original SDQ, in our indicator system, the higher the scores of these five subscales, the more serious the corresponding psychological problems.^3^ Furthermore, we can obtain a comprehensive indicator *Psychological problems* by adding up the scores of each subscale to measure the overall status of adolescent mental health.

#### The shock of the COVID-19 pandemic

In terms of the core explanatory variable, we referred to the DID method, and divided the samples into treatment group and control group by the cumulative number of confirmed cases of COVID-19 in each city in the first half of 2020. First of all, it is important to note that COVID-19 mainly spread intensively in China in the first half of 2020, and the situation has gradually recovered in the second half of the year. Meanwhile, the data of the DYH in 2020/2021 was also investigated in the second half of 2020. Therefore, we used the cumulative number of confirmed cases in each city in the first half of 2020 to examine the impact of COVID-19, which is also more reasonable. According to the COVID-19 Timely Dynamic Tracking Database, from January 20 to June 30 in 2020, among the cities in the sample, Jinan, Weifang, Jining, Weihai, Dezhou, Liaocheng, Linyi, and Heze all had confirmed cases, but there were no confirmed cases in Dongying and Laiwu (see Table A2 in the Appendix). Therefore, we construct the binary shock variable *COVID19*. Specifically, as shown in Table A2, when the observation is affected by the COVID-19 pandemic, the core explanatory variable, COVID-19*Post, is equal to 1, including students in Jinan, Weifang, Jining, Weihai, Dezhou, Liaocheng, Linyi, Heze in 2020. If the observations were not or barely affected by the COVID-19 pandemic, it is assigned to 0, including students in Dongying and Laiwu in 2020, and samples in 2017.

#### Control variables

As shown in Table 1, we control a series of characteristic variables about students and their families, including students’ gender, age, *Hukou*,^4^ migrant, elite class, health, schooling of father and mother, family wealth, etc. Adolescent mental health may be associated with some characteristics at the individual and family levels, so controlling for student and family-level characteristics as much as possible can help minimize potential endogeneity problems. The definitions and summary statistics of variables in our research are reported in Table 1.

**Table 1:** Variable definition and summary statistics.

Variable	Definition	*N*	Mean	S.D.
**Panel A.** Indicators of psychological problems (SDQ method)				
Psychological problems	Degree of student psychological problems as measured by SDQ method (0–60 points)	12,096	8.579	10.270
Emotional symptoms	Subscale of SDQ (0–12 points)	12,096	2.058	2.647
Conduct problems	Subscale of SDQ (0–12 points)	12,096	1.590	2.069
Inattention	Subscale of SDQ (0–12 points)	12,096	1.835	2.366
Peer relationship problems	Subscale of SDQ (0–12 points)	12,096	1.529	2.135
Prosocial problems	Subscale of SDQ (0–12 points)	12,096	1.567	2.173
**Panel B.** COVID-19 shock				
COVID19*Post	Shock variable of DID. 1 = adolescent in cities hit by COVID-19 in 2020; 0 = surveying in 2017 or adolescent in cities barely affected by the COVID-19 in 2020	12,096	0.608	0.488
**Panel C.** Control variables				
Gender	Girls = 0; boys = 1	12,096	0.481	0.500
Age	Adolescent age (years old)	12,096	16.21	1.099
*Hukou*	Rural = 0; Urban = 1	12,096	0.454	0.498
Migrant	Whether the student is a migrant child (no = 0; yes = 1)	12,096	0.083	0.276
Elite class	Whether the student is in an elite class (no = 0; yes = 1)	12,096	0.193	0.394
Health	Adolescent physical health status through self-assessment (1 = poor; 2 = general; 3 = good; 4 = very good; 5 = excellent)	12,096	3.546	1.043
Only child	Whether the student is an only child (no = 0; yes = 1) 1 = unschooled; 2 = primary school; 3 = junior middle school; 4 = specialized secondary school; 5 = vocational high school; 6 = senior high school; 7 = junior college; 8 = bachelor degree; 9 = master’s or Ph.D. degree	12,096	0.323	0.468
Schooling of father		12,096	4.338	1.988
Schooling of mother	1 = unschooled; 2 = primary school; 3 = junior middle school; 4 = specialized secondary school; 5 = vocational high school; 6 = senior high school; 7 = junior college; 8 = bachelor degree; 9 = master’s or Ph.D. degree	12,096	3.949	1.999
Father’s occupation	1 = the adolescent father is a national civil servant; enterprise manager; teachers, engineers, doctors, lawyers and other high-tech occupations; 0 = other occupations	12,096	0.168	0.374
Wealth	Relatively household wealth (1–5 from low to high)	12,096	2.891	0.618
Internet	whether there is a computer at home with Internet access (no = 0; yes = 1)	12,096	0.859	0.348
Board	Boarding at the school from Monday to Thursday (no = 0; yes = 1)	12,096	0.715	0.451

### Methodology

Unlike previous related studies that relied on small-scale questionnaires, our study is based on large-scale micro-survey data and is based on a socio-economic perspective. Therefore, we examine the outbreak of the COVID-19 pandemic as a quasi-natural experiment and used the DID model to identify the causal effects of the COVID-19 pandemic on adolescent mental health, which is a widely employed approach to evaluate the impact of a shock caused by epidemic disease or natural disasters (Domino et al., 2003; Mu and Chen, 2016). In addition, there is certain rationality and validity in using the DID method in our research. Specifically, firstly, the intensive spread of COVID-19 in China was in the first half of 2020, and DYH included both the mental health data of students in the 2017/2018 and 2020/2021 academic years,^5^ that is, the period before and after the occurrence of the COVID-19 pandemic. Secondly, among the cities investigated, there were no confirmed cases of COVID-19 in Dongying and Laiwu in the first half of 2020, while other cities all had confirmed cases, which provides a good control group for the DID model. Therefore, based on the principles of the DID method, we can identify the causal effect of the COVID-19 pandemic on adolescent mental health by comparing the changes in mental health status of sampled students from the treatment group and control group, before and after the outbreak of the COVID-19 pandemic.

The DID model is one of the classic methods to evaluate the effect of exogenous shocks, events, or policies, which is widely employed in social science (e.g., O’Connell et al., 2022; Zhao et al., 2022; Zhao and Yan, 2022). When evaluating the effects of a shock, such as the COVID-19 pandemic, if we just simply compare the difference of the dependent variable before and after the shock, the short-term trend changes may interfere with the estimation results and lead to the causality effects being uncertain. However, in the DID identification strategy, the before-and-after change of the dependent variables in the control group is used as an approximation for the short-term trend changes. Specifically, we regarded those students who were barely affected by COVID-19 in 2020 as the control group, and estimate this short-term trend changes. Then, the DID estimate can be obtained by subtracting the short-term trend changes from the before-and-after change of the dependent variables in the treatment group, which consists of students who were already affected by COVID-19 in 2020. The empirical model is set up as follows:


(1) $${MH}_{ict}=\beta_{0}+\beta_{1}\mathrm{COVID}19_{c}*{\mathrm{Post}}_{t}+\beta_{2}X_{ict}+\gamma_{c}+\theta_{t}+\varepsilon_{ict}$$

In the equation (1), the subscript i accounts for student, c denotes city, and t refers to year. ${MH}_{ict}$ indicates the mental health status (psychological problems) of the student i in the city c in the year t. $\mathrm{COVID}19_{c}$ represents whether the city c was affected by the spread of COVID-19. ${\mathrm{Post}}_{t}$ is a dummy that equals to 1 indicating that the survey year t is after the COVID-19 shock, and 0 otherwise. $\beta_{1}$ is the DID estimate, representing the effect of the COVID-19 pandemic on adolescent mental health, which is the key coefficient we are interested in. If $\beta_{1}$ is significantly positive, it means that the COVID-19 pandemic leads to an increase in psychological problems among adolescents and worsens their mental health.$X_{ict}$ denotes a series of control variables, as mentioned above. Furthermore, we control the city fixed effect $\gamma_{c}$ and year fixed effect $\theta_{t}$, which are used to control for city characteristics and time trends in the framework of DID methods.^6^ $\varepsilon_{ict}$ is the random error term.

## Results

### The impact of the COVID-19 pandemic on adolescent mental health

Table 2 reports the baseline results of the COVID-19 pandemic on adolescent psychological problems. In column (1), we find that the shock of COVID-19 significantly increased the scores of adolescents’ psychological problems by an average of 2.5677 points, which is about 29.93% of the mean value of this explained variable, *Psychological problems*. Thus, similar to the literature in medicine and public health (e.g., Duan et al., 2020; Qi et al., 2020; Chi et al., 2021; Qu et al., 2021; Cao et al., 2022), by using the DID method, we also confirm that the COVID-19 pandemic significantly increased adolescent psychological problems, and had a significant negative impact on their overall mental health.

**Table 2:** The impact of COVID-19 on student mental health.

	(1)	(2)	(3)	(4)	(5)	(6)
	Psychological Problems	Emotional symptoms	Conduct problems	Inattention	Peer relationship problems	Prosocial problems
COVID19*Post	2.5677^***^	0.5733^***^	0.4655^***^	0.5028^***^	0.4406^***^	0.5854^***^
	(0.6453)	(0.1539)	(0.1300)	(0.1424)	(0.1357)	(0.1341)
	[1.30, 3.83]	[0.27, 0.87]	[0.21, 0.72]	[0.22, 0.78]	[0.17, 0.71]	[0.32, 0.85]
Gender	0.1343	−0.1241^***^	−0.0269	0.0036	0.1426^***^	0.1392^***^
	(0.1817)	(0.0471)	(0.0371)	(0.0424)	(0.0385)	(0.0389)
Age	−0.0541	−0.0068	−0.0144	0.0071	−0.0192	−0.0207
	(0.0820)	(0.0211)	(0.0168)	(0.0191)	(0.0173)	(0.0175)
*Hukou*	−0.3490	−0.0707	−0.1141^**^	−0.0415	−0.0463	−0.0763
	(0.2320)	(0.0626)	(0.0471)	(0.0540)	(0.0490)	(0.0500)
Migrant	−0.0930	0.0253	0.0168	−0.0689	−0.0148	−0.0514
	(0.3073)	(0.0827)	(0.0616)	(0.0718)	(0.0662)	(0.0662)
Elite class	0.2026	0.0053	0.0522	0.0292	0.0726	0.0434
	(0.2354)	(0.0597)	(0.0489)	(0.0547)	(0.0502)	(0.0509)
Health	−2.6853^***^	−0.6871^***^	−0.4575^***^	−0.5992^***^	−0.4634^***^	−0.4781^***^
	(0.0976)	(0.0257)	(0.0197)	(0.0226)	(0.0206)	(0.0209)
Only child	0.5045^**^	0.0917	0.1356^***^	0.0484	0.1152^**^	0.1136^**^
	(0.2215)	(0.0572)	(0.0452)	(0.0506)	(0.0475)	(0.0474)
Schooling of father	−0.2886^***^	−0.0666^***^	−0.0492^***^	−0.0604^***^	−0.0526^***^	−0.0598^***^
	(0.0653)	(0.0168)	(0.0137)	(0.0149)	(0.0139)	(0.0142)
Schooling of mother	−0.1183^*^	−0.0316^*^	−0.0184	−0.0187	−0.0245^*^	−0.0251^*^
	(0.0625)	(0.0163)	(0.0132)	(0.0143)	(0.0133)	(0.0136)
Father’s occupation	0.8980^***^	0.1119	0.2452^***^	0.1479^**^	0.1855^***^	0.2075^***^
	(0.2885)	(0.0717)	(0.0617)	(0.0663)	(0.0611)	(0.0617)
Wealth	−0.8606^***^	−0.2120^***^	−0.1564^***^	−0.2007^***^	−0.1378^***^	−0.1536^***^
	(0.1845)	(0.0455)	(0.0388)	(0.0427)	(0.0387)	(0.0392)
Internet	−1.7229^***^	−0.2833^***^	−0.3733^***^	−0.3619^***^	−0.3354^***^	−0.3690^***^
	(0.2993)	(0.0740)	(0.0614)	(0.0686)	(0.0642)	(0.0643)
Board	0.0413	−0.0483	0.0060	−0.0083	0.0499	0.0419
	(0.2125)	(0.0557)	(0.0438)	(0.0493)	(0.0447)	(0.0456)
City fixed effect	Yes	Yes	Yes	Yes	Yes	Yes
Year fixed effect	Yes	Yes	Yes	Yes	Yes	Yes
Constant	23.8722^***^	5.7789^***^	4.3896^***^	4.9493^***^	4.3345^***^	4.4198^***^
	(1.5650)	(0.4033)	(0.3206)	(0.3613)	(0.3297)	(0.3354)
*R*-squared	0.1151	0.1030	0.0965	0.1038	0.0832	0.0886
*N*	12,096	12,096	12,096	12,096	12,096	12,096

^***^, ^**^, ^*^ indicate significance at the levels of 1, 5, and 10%, respectively. Robust standard errors are presented in parentheses. 95% confidence intervals for COVID19^*^Post are reported in brackets. Only the confidence intervals for the key explanatory variable COVID19^*^Post are reported in the table, while confidence intervals for the remaining variables can be calculated from the estimated coefficients and standard errors.

Further, in columns (2) to (6), we examined the impacts of the COVID-19 pandemic on each subscale of mental health, including *Emotional symptoms*, *Conduct problems*, *Inattention*, *Peer relationship problems*, and *Prosocial problems*. As mentioned earlier, these 5 subscales are scored on a scale of 0–12, and the higher the score, the more serious the corresponding psychological problem. According to these results in Table 2, we find that all coefficients on COVID19*Post are significantly positive at the 1% level, suggesting that the COVID-19 pandemic also significantly increased the scores of the 5 subscales. Therefore, the negative impacts of the COVID-19 on adolescent mental health may be comprehensive, and manifested in various aspects.

More specifically, as shown in columns (2), (4), and (5), the COVID-19 pandemic greatly increased the severity of adolescent negative emotions and directly hindered their prosocial tendencies and peer relationships. Moreover, the results of columns (3) and (4) tell us that the COVID-19 had a negative impact on adolescent behaviors, such as making teenagers more easily to be angry, accusing others, and being easily distracted. Meanwhile, it is worth noting that many studies have reached similar conclusions using data from other countries (Patra and Patro, 2020; Hu and Qian, 2021; Idele and Banati, 2021; Meyers et al., 2021). Our evidence indicates that the negative impact of the COVID-19 pandemic on adolescent mental health is widespread globally, and this issue needs more attention, especially in developing countries.

### Mechanism tests

In “Theoretical mechanisms and research hypotheses,” we theoretically identify three mechanisms by which the COVID-19 pandemic affects adolescent mental health, namely social trust, digital divide, and family income gap. We now further test these mechanisms and examine the hypotheses.

#### Social trust

The COVID-19 pandemic may negatively impact adolescent mental health by reducing their social trust, especially with strangers in public. As mentioned in Hypothesis 1, negative public health events such as the COVID-19 pandemic have posed a huge challenge to social trust (Kye and Hwang, 2020; Woelfert and Kunst, 2020; Aassve et al., 2021), especially for adolescents. Based on the relevant questions in the questionnaire, we examined the impact of the COVID-19 pandemic on adolescents’ social trust from three aspects: taking public transportation, appearing in crowded places, and eating in public places. The reason we construct social trust indicators from these three dimensions is that in these scenarios, adolescents have to come into contact with strangers. During the COVID-19 pandemic, these variables can partially reflect adolescent social trust.

In Table 3, we find that the coefficients on COVID19*Post are all significantly positive in three columns. These results suggest that the shock of the COVID-19 pandemic not only significantly increased adolescents’ fear of taking public transportation, but also increased their discomfort in crowded public places and eating in public places. Thus, our estimates imply that the COVID-19 pandemic further worsens adolescent mental health by reducing their social trust, which supports Hypothesis 1.

**Table 3:** Mechanisms: COVID-19 and social trust.

	(1)	(2)	(3)
	Fear of taking public transport	Feeling uncomfortable in crowded public places	Feeling uncomfortable eating in public
COVID19^*^Post	0.2731^***^	0.1416^***^	0.0957^*^
	(0.0474)	(0.0523)	(0.0537)
	[0.18, 0.37]	[0.04, 0.24]	[−0.01, 0.20]
Control variables	Yes	Yes	Yes
City fixed effect	Yes	Yes	Yes
Year fixed effect	Yes	Yes	Yes
Constant	2.0757^***^	2.5691^***^	2.4143^***^
	(0.1211)	(0.1393)	(0.1419)
R-squared	0.0441	0.0622	0.0506
N	12,096	12,096	12,096

^***^, ^**^, ^*^ indicate significance at the levels of 1, 5, and 10%, respectively. Robust standard errors are presented in parentheses. The above three mechanistic variables Fear of taking public transport, Feeling uncomfortable in crowded public places, and Feeling uncomfortable eating in public are all scored from 1 to 5. The higher the score, the higher the degree of panic or discomfort. 95% confidence intervals for COVID19^*^Post are reported in brackets.

#### Digital divide

In “Theoretical mechanisms and research hypotheses,” we have highlighted the COVID-19 pandemic may further widen the digital divide between different households, adversely affecting the mental health of some adolescents. To test Hypothesis 2, we construct the interaction term of the COVID-19 pandemic and Internet accessibility (COVID19*Post*Internet), and estimate this marginal effect on adolescent mental health.

By using the DID model, the results are represented in column (1) of Table 4, we find that the coefficient on this interaction is −1.0688, with significance statistical significance at the 10% level. These results suggest that during the COVID-19 pandemic, the Internet can further alleviate adolescent psychological problems compared with adolescents without the Internet, as Internet access can reduce the psychological problems in adolescents in Tables 2, 4. Therefore, our estimates provide positive evidence for Hypothesis 2 that the shock of the COVID-19 pandemic has clearly widened the inequality created by the digital divide, with a stronger negative impact on the mental health of some adolescents with fewer digital resources.

**Table 4:** Mechanisms: COVID-19 and digital divide and income gap.

	(1)	(2)	(3)	(4)
	Digital divide	Income gap	Considering both digital divide and income gap	Only low-income families
COVID19^*^Post	3.5115^***^	2.2944^***^	2.9047^***^	4.2020^**^
	(0.8323)	(0.6458)	(0.8367)	(1.7612)
	[1.88, 5.14]	[1.03, 3.56]	[1.26, 4.54]	[0.75, 7.66]
Internet	−1.1234^**^		−1.1685^**^	0.4345
	(0.4629)		(0.4680)	(0.8875)
	[−2.03, −0.22]		[−2.09, −0.25]	[−1.31, 2.17]
COVID19^*^Post^*^Internet	−1.0688^*^		−0.6667	−2.2448^*^
	(0.6004)		(0.6043)	(1.1714)
	[−2.25, 0.11]		[−1.85, 0.52]	[−4.54, 0.05]
Low-income family		0.9547^*^	1.0265^*^	
		(0.5315)	(0.5338)	
		[−0.09, 2.00]	[−0.02, 2.07]	
COVID19^*^Post^*^Low-income family		1.7213^***^	1.5824^***^	
		(0.5542)	(0.5585)	
		[0.64, 2.81]	[0.49, 2.68]	
Control variables	Yes	Yes	Yes	Yes
City fixed effect	Yes	Yes	Yes	Yes
Year fixed effect	Yes	Yes	Yes	Yes
R-squared	0.1153	0.1176	0.1196	0.1130
N	12,096	12,096	12,096	2017

^***^, ^**^, ^*^ indicate significance at the levels of 1, 5, and 10%, respectively. Robust standard errors are presented in parentheses. Low-income family is a dummy variable, if the value is 1, it means that the family is relatively poor or very poor in the self-assessment. COVID-19^*^ Internet and COVID-19^*^ Low-income family represent the interaction terms of COVID-19 and Internet and Low-income family, respectively. 95% confidence intervals are reported in brackets.

#### Family income gap

In Hypothesis 3, we point out that the widening family income gap due to the COVID-19 pandemic may further worsen the mental health of some adolescents. We divided the full sample into three groups: low-income, middle-income, and high-income families,^7^ and constructed the interaction term of the COVID-19 pandemic and low-income family (COVID19*Post*Low-income family) for the DID estimations. In addition, it is important to highlight that even though there is some correlation between the digital divide and the family income gap, *Low-income family* in the study specifically refers to relatively low-income family, not poor family, and thus not all relatively low-income families in the sample do not have access to the Internet. Also, the digital divide focuses on the prevention of the epidemic through digital technology and the online education for students, while the family income gap focuses more on the increase of inequality in family income and access to necessities during the COVID-19 pandemic. The two mechanisms have distinct differences in perspective and therefore need to be discussed separately.

In column (2) of Table 4, not surprisingly, the coefficient of the variable *Low-income family* is significantly positive, implying that teenagers from low-income families are more likely to have psychological problems than those from middle-income and high-income families. Moreover, the coefficient of COVID19*Post*Low-income family keeps significantly positive, indicating that the COVID-19 pandemic has further increased the occurrence and severity of psychological problems among adolescents from low-income families, confirming the accuracy of our previous research Hypothesis 3.

#### Considering both digital divide and family income gap

As shown in Table A3 in the Appendix, there are 671 adolescents in the sample who are both from low-income families and also do not have Internet access at home, which means that about 30.59% of the low-income families (2017) do not have Internet. Therefore, we need to further test whether there is a potential link between the two mechanisms of digital divide and family income gap. In column (3) of Table 4, we consider both digital divide and family income gap in a model, and find that the coefficient of COVID19*Post*Low-income family remains significant at the 1% statistical level, but the coefficient of COVID19*Post*Internet is not significant.

The main reason for this coefficient change may be that in the model of column (1), some families without Internet are also low-income, and when only the moderating effect of Internet is considered (i.e., when *Low-income family* and COVID19*Post*Low-income family are not controlled), the deterioration of mental health due to lower income in this part of the sample is ignored, thus overestimating the moderating effect of Internet. In addition, since the coefficient of COVID19*Post*Internet is only statistically significant at the 10% level in column (1), its significance is not strong, and thus tends to be weakened when controlling for both moderating effects of digital divide and family income gap.

Furthermore, in column (4) of Table 4, we retain only the low-income families, which completely exclude the difference between low-income and middle or high-income families, to verify the moderating effect of the Internet access more clearly. The results show that among the low-income families, the coefficient of COVID19*Post*Internet is −2.2448 and statistically significant at the 10% level, which indicates that even in low-income families, Internet access still significantly weakens the negative impact of the COVID-19 pandemic on adolescent mental health. Therefore, based on the results in columns (1) to (4) of Table 4, we can confirm that although there is some association between the two moderating effects of digital divide and family income gap, the effects are not exactly parallel.

### Heterogeneity analysis

We explore the heterogeneity of the COVID-19 pandemic on adolescent mental health from three perspectives: gender, only children, and *hukou*.

First, we analyze the heterogeneity effect by gender. In columns (1) and (2) of Table 5, we find that the COVID-19 pandemic intensifies psychological problems for both boys and girls, with a slightly higher significance for boys. This subtle difference may be due to boys being more prone to social relationship problems and prosocial problems (Gore et al., 1993; Fisher and Ryan, 2021). Specifically, as reported in columns (5) and (6) in Table 2, although gender did not significantly influence the occurrence or aggravation of psychological problems, boys were more likely to have peer relationship problems and prosocial problems than girls. In the mechanistic analysis, the shock of COVID-19 pandemic negatively affected the adolescent social distance and social trust, and the above factors may have more influence on the 2 subscales of peer relationship and prosocial tendency, with boys already performing worse. Therefore, we believe that the depressing effects of COVID-19 on mental health may be more pronounced for boys.

**Table 5:** Heterogeneity due to student and family characteristics.

	(1)	(2)	(3)	(4)	(5)	(6)
	Girls	Boys	Only child	Non-only child	Urban	Rural
COVID-19^*^Post	2.0653^**^	3.3648^***^	2.6753^**^	2.5001^***^	2.4463^**^	1.9624^**^
	(0.8397)	(0.9960)	(1.1000)	(0.7934)	(1.0190)	(0.8369)
	[0.42, 3.71]	[1.41, 5.32]	[0.52, 4.83]	[0.95, 4.06]	[0.45, 4.44]	[0.32, 3.60]
Control variables	Yes	Yes	Yes	Yes	Yes	Yes
City fixed effect	Yes	Yes	Yes	Yes	Yes	Yes
Year fixed effect	Yes	Yes	Yes	Yes	Yes	Yes
R-squared	0.0989	0.1368	0.1148	0.1189	0.1407	0.1055
N	6,274	5,822	3,907	8,189	6,160	5,936

^***^, ^**^, ^*^ indicate significance at the levels of 1, 5, and 10%, respectively. Robust standard errors are presented in parentheses. 95% confidence intervals for COVID-19^*^Post are reported in brackets.

Second, the results in columns (3) and (4) of Table 5 show that the adverse effects of the COVID-19 pandemic on the mental health of both non-only and non-only children are significant. However, this difference is not obvious from the estimated results. Thus, in terms of the impact of COVID-19 on adolescent mental health, the heterogeneous effects of the negative impact are not known for either only children who may enjoy more family resources or non-only children who have more siblings with them.

Third, we consider urban–rural heterogeneity. In this paper, urban areas mainly include the city center and counties, while rural areas contain the towns and villages. According to the results of columns (5) and (6) in Table 5, the mental health of adolescents living in both urban and rural areas was negatively affected by the COVID-19 pandemic. In contrast, the mental health of urban adolescents was more affected by the pandemic, probably because the COVID-19 pandemic spread more widely in urban China and the corresponding prevention and control measures were more stringent in urban areas. Taken together, based on the heterogeneity analysis, it is clear that the adverse impact of the COVID-19 pandemic on the mental health of Chinese adolescents is widespread.

### Robustness checks

To ensure the robustness of the estimated results, we conducted a series of robustness checks, including placebo test, excluding some extreme samples, and adding control variables.

First, in column (1) of Table 6, we perform a placebo test by advancing the COVID-19 shock, which is widely used in robustness checks of DID estimations (Chen and Zhao, 2022; Zhao and Yan, 2022). Specifically, we simulate the COVID-19 shock forward by one period, which is the 2017/2018 academic year based on our data feasibility. Then we construct the variable COVID19*Post_1. The results show that the coefficient on this pseudo-variable changes greatly compared with the baseline results in Table 2. Thus, the placebo test indicates that the pseudo effect we construct does not exist, which indirectly verifies the reliability of the baseline results.

**Table 6:** Robustness checks.

	(1)	(2)	(3)	(4)	(5)	(6)
	Placebo test of advancing the shock by one period	Excluding very poor and very wealthy households	Excluding students with poor physical health	Excluding students whose father or mother (or both) received tertiary education	Excluding students whose father or mother (or both) is a teacher, doctor, or other professional technician	Additional control variables
COVID-19^*^Post		2.4017^***^	2.3981^***^	2.2440^***^	2.5326^***^	2.1030^***^
		(0.6517)	(0.6401)	(0.7224)	(0.6838)	(0.6430)
		[1.12, 3.68]	[1.14, 3.65]	[0.83, 3.66]	[1.19, 3.87]	[0.84, 3.36]
COVID-19^*^Post_1	−0.8067^*^					
	(0.4145)					
	[−1.62, 0.01]					
Control variables	Yes	Yes	Yes	Yes	Yes	Yes
Additional control variables	No	No	No	No	No	Yes
City fixed effect	Yes	Yes	Yes	Yes	Yes	Yes
Year fixed effect	Yes	Yes	Yes	Yes	Yes	Yes
*R*-squared	0.1135	0.1145	0.1008	0.1104	0.1127	0.1331
*N*	12,096	11,487	11,865	9,541	10,909	12,096

^***^, ^**^, ^*^ indicate significance at the levels of 1, 5, and 10%, respectively. The explained variable in all columns is Psychological problems. Robust standard errors are presented in parentheses. Post_1 represents that the variable Post advances one period. 95% confidence intervals are reported in brackets.

Second, we adopt the method of eliminating extreme observations to ensure the generality of the samples. Socioeconomic status is an important factor affecting adolescent mental health (Wildman, 2003; Reiss, 2013), and adolescents from relatively poor and wealthy families may have differences in their own mental health from most samples. Thus, we eliminate adolescents from very poor and very wealthy households in column (2) in Table 6. In addition, given the strong association between physical and mental health (Feiss and Pangelinan, 2021), we exclude adolescents who self-rated poor physical health in column (3). Moreover, parents’ education and occupation are likely to affect students’ mental health. Thus, we remove adolescents whose parents are highly educated, or engaged in highly skilled occupations^8^ in columns (4) and (5), respectively. Clearly, the results are also basically consistent with the baseline results in Table 2.

Third, we add some additional control variables to further mitigate potential endogeneity issues in column (6) of Table 6, including student-parent relationship, parental education expectations, parents’ attitudes towards their children’s education, students’ participation in extracurricular education, and awards in school. Table A4 in the Appendix reports the definitions and summary statistics of these variables. We find that the coefficient and significance of COVID19*Post are virtually unchanged.

## Discussion and conclusion

The potential impacts of the COVID-19 pandemic on adolescent mental health have attracted considerable interest mainly in the fields of medical and public health (e.g., Racine et al., 2020; Xiang et al., 2020; Hu and Qian, 2021; Shek et al., 2021; Wright et al., 2021; Kim et al., 2022). Most of these studies were mainly based on online questionnaires and small-scale survey data, and analyzed through psychological and medical perspectives (Guessoum et al., 2020), while few studies used large-scale social survey data to explore this issue from a socioeconomic perspective (Banati et al., 2020; Brodeur et al., 2021; Jaspal and Breakwell, 2022). In addition, by using data from adolescents in different countries, such as China (e.g., Chen et al., 2020; Cao et al., 2022), Germany (Ravens-Sieberer et al., 2022), India (e.g., Patra and Patro, 2020), Italy (e.g., Commodari and La Rosa, 2020), the United Kingdom (e.g., Hu and Qian, 2021; Wright et al., 2021), the United States (e.g., Rogers et al., 2021) etc., scholars have observed a trend of deterioration in adolescent mental health and an increase in the frequency of psychological problems during the COVID-19 period (Adibelli and Sümen, 2020; Shek et al., 2021; Wu et al., 2021; Cao et al., 2022). Based on a large-scale social survey, the DYH in China, we adopt the DID model to identify the impact of the COVID-19 pandemic on adolescent mental health and explore the socioeconomic mechanisms. Unlike previous studies that have focused specifically on psychological and behavioral changes in adolescents, our study places greater emphasis on social trust among adolescents and the exacerbation of inequalities during the COVID-19 pandemic, which adds to the existing literature and provides new insights.

We draw several conclusions from our findings. First, we find that the COVID-19 pandemic has significantly increased adolescent psychological problems in China. Worse, the negative impact of the COVID-19 pandemic on adolescent mental health is very comprehensive in terms of each subscale, including emotional symptoms, conduct problems, inattention, peer relationship problems, and prosocial problems. Specifically, COVID-19 pandemic significantly increased the scores of adolescents’ psychological problems by an average of 2.5677 points, which corresponds to an average decrease of 29.93% in mental health. Second, we explore several socioeconomic mechanisms, indicating that the negative impact of the COVID-19 pandemic on adolescent mental health can be explained by a reduction in adolescents’ social trust, and widening inequalities caused by the digital divide and income gap. Third, the results of heterogeneity analysis suggest that the COVID-19 pandemic has a greater negative influence on the mental health of boys and urban adolescents.

Our findings offer some insights into preventing adolescent psychological problems in China and other countries during the COVID-19 pandemic. The Chinese government has adopted strict pandemic prevention and control measures, such as the dynamic zero-COVID policy, to minimize the negative socioeconomic impacts of the COVID-19 pandemic as soon as possible. However, even so, adolescent mental health is inevitably negatively impacted by the pandemic (Zhang et al., 2020; Qu et al., 2021; Cao et al., 2022). According to the findings of our research, the decline in social trust of adolescents caused by the COVID-19 pandemic is one of the important mechanisms for the deterioration of their mental health. Therefore, it is recommended that the government continue to take active pandemic prevention measures to avoid the spread of the epidemic as much as possible, thereby enhancing adolescent social trust and making them feel safer in public.

In addition, we provide evidence that the shock of the COVID-19 pandemic has exacerbated the inequalities caused by the digital divide and income gap in China, and the mental health of adolescents from families with low income and fewer digital resources has further deteriorated during the pandemic. Thus, to enhance the mental health of disadvantaged adolescents and promote human capital enhancement, it is suggested that the government, schools, public welfare funds and other relevant institutions provide them with more economic support and social care.

There are some limitations of this paper and recommendations for further research. First, the data used in this study only covers cities in Shandong Province, China; thus, there are certain limitations in the scope of the sample. Second, limited by the database, we only discuss and test the socioeconomic mechanisms from three aspects: social trust, digital divide, and income gap, and more potential mechanisms cannot be further confirmed. Third, the database used in this study only investigated the mental health of senior high school students, so the impact of the COVID-19 pandemic on adolescents at other educational phases remains to be explored.

## Data availability statement

The original contributions presented in the study are included in the article/Supplementary material, further inquiries can be directed to the corresponding author.

## Author contributions

BC: data curation, conceptualization, software, methodology, writing-original draft, writing—reviewing and editing, and funding acquisition. CZ: conceptualization, methodology, validation, writing—original draft, and funding acquisition. XL: validation, formal analysis, and writing—reviewing and editing. JL: conceptualization and writing—reviewing and editing. All authors contributed to the article and approved the submitted version.

## Funding

This work was supported by the Fundamental Research Funds for the Central Universities (Nos. QCDC-2020-10 and QCDC-2020-21), and the Tsinghua Rural Studies PhD Scholarship (202110).

## Conflict of interest

The authors declare that the research was conducted in the absence of any commercial or financial relationships that could be construed as a potential conflict of interest.

## Publisher’s note

All claims expressed in this article are solely those of the authors and do not necessarily represent those of their affiliated organizations, or those of the publisher, the editors and the reviewers. Any product that may be evaluated in this article, or claim that may be made by its manufacturer, is not guaranteed or endorsed by the publisher.

## Footnotes

^1^Data source: https://news.sina.cn/zt_d/yiqing0121?vt=4.

^2^For more information on the Strengths and Difficulties Questionnaire (SDQ), see www.sdqinfo.org.

^3^In the original SDQ, a higher score indicates the better mental health for the prosocial behavior subscale, while it indicates worse mental health for the prosocial behavior in our measurement. The advantage of this approach is that it is easier for us obtain a comprehensive indicator of mental health (*Psychological problems*) by adding up the scores of 5 subscales.

^4^The household registration (*Hukou*) system of China is a household-based population management policy implemented in China. In the *Hukou* system, the Hukou status of the population is divided according to their location and family members, and can be mainly divided into two categories: rural *Hukou* and urban *Hukou*.

^5^The 2020/2021 academic year starts in the second half of 2020.

^6^Controlling for city and year fixed effects allows us to use a reduce d form of the DID model, eliminating the need for additional controls for COVID-19 and Post.

^7^The DHY surveyed the self-rated household economic situation. The relevant variable ranges from 1–5, where 1 and 2 indicate low income, 3 denotes middle income, and 4 and 5 mean high income.

^8^Here, one of the parents is a teacher, a doctor or other technical jobs, which are regarded as special samples.
