# Supplementary material for: COVID-19 pandemic and adolescent mental health in China: Micro evidence and socioeconomic mechanisms
Source: Front Psychol. 2022 Nov 14;13:1041376. doi: 10.3389/fpsyg.2022.1041376 (PMC9703070; doi:10.3389/fpsyg.2022.1041376)
Supplement: Supplementary file 1 [file Table_1.docx]

**Appendix**

**Table A1.** The measurement of students’ mental health through the SDQ

| Indicators | Assignments |
| --- | --- |
| **Panel A.** Subscales of SDQ | |
| Emotional symptoms  (0-12 points) | Feeling lonely (0 = never; 1 = not serious; 2 = moderate; 3 = relatively serious; 4 = very serious)  Feeling worried (0 = never; 1 = not serious; 2 = moderate; 3 = relatively serious; 4 = very serious)  Feeling scared (0 = never; 1 = not serious; 2 = moderate; 3 = relatively serious; 4 = very serious) |
| Conduct problems  (0-12 points) | Getting very angry and often lose the temper (0 = never; 1 = not serious; 2 = moderate; 3 = relatively serious; 4 = very serious)  Getting a lot of stomach-aches or sickness (0 = never; 1 = not serious; 2 = moderate; 3 = relatively serious; 4 = very serious)  Blaming others frequently (0 = never; 1 = not serious; 2 = moderate; 3 = relatively serious; 4 = very serious) |
| Inattention  (0-12 points) | Being easily distracted, and finding it difficult to concentrate (0 = never; 1 = not serious; 2 = moderate; 3 = relatively serious; 4 = very serious)  Being restless, and cannot stay still for long (0 = never; 1 = not serious; 2 = moderate; 3 = relatively serious; 4 = very serious)  The Mind goes blank (0 = never; 1 = not serious; 2 = moderate; 3 = relatively serious; 4 = very serious) |
| Peer relationship problems  (0-12 points) | Feeling that people are not friendly to you and don’t like you (0 = never; 1 = not serious; 2 = moderate; 3 = relatively serious; 4 = very serious)  Arguing with people around you frequently (0 = never; 1 = not serious; 2 = moderate; 3 = relatively serious; 4 = very serious)  Feeling that people around you are not close (0 = never; 1 = not serious; 2 = moderate; 3 = relatively serious; 4 = very serious) |
| Prosocial problems  (0-12 points) | Feeling that most people in the society cannot be trusted (0 = never; 1 = not serious; 2 = moderate; 3 = relatively serious; 4 = very serious)  Feeling that others are trying to take advantage of you (0 = never; 1 = not serious; 2 = moderate; 3 = relatively serious; 4 = very serious)  Feeling that others don’t understand you, or don’t sympathize with you (0 = never; 1 = not serious; 2 = moderate; 3 = relatively serious; 4 = very serious) |
| **Panel B.** Comprehensive indicator of psychological problems | |
| Psychological problems  (0-60 points) | The comprehensive indicator of psychological problems is obtained by summing the scores of the above five subscales of SDQ. The higher the score, the more serious the psychological problem of the student |

Notes: Strengths and Difficulties Questionnaire (SDQ) is an internationally recognized self-assessment questionnaire designed to measure student mental health. The scores of each question in the table are also self-assessed by the students under investigation.

**Table A2.** Treatment group and control group

|  | Number of observations | Percentage（%） | Cumulative number of confirmed cases  (From Jan. 20 to Jun. 30 in 2020) |
| --- | --- | --- | --- |
| **Panel A.** Wave |  |  |  |
| 2017/2018 | 3168 | 26.19 | -- |
| 2020/2021 | 8928 | 73.81 | -- |
| **Panel B.** Cities in the sample  **Treatment group** (Cities where COVID-19 has spread in 2020) | | | |
| Jinan | 698 | 5.77 | 47 |
| Weifang | 667 | 5.51 | 44 |
| Jining | 1988 | 16.44 | 260 |
| Weihai | 1179 | 9.75 | 38 |
| Dezhou | 1580 | 13.06 | 37 |
| Liaocheng | 1439 | 11.90 | 38 |
| Linyi | 1412 | 11.67 | 49 |
| Heze | 975 | 8.06 | 18 |
| **Control group** (Cities with no confirmed cases of COVID-19 in 2020 and observations in 2017) | | | |
| Dongying | 1066 | 8.81 | 0 |
| Laiwu | 1092 | 9.03 | 0 |
| Observations in 2017 | 3168 | 26.19 | -- |

Notes: Since the spread of COVID-19 in China was mainly concentrated in the first half of 2020, and the investigation was conducted in the second half of 2020, the study differentiated the treatment group and the control group based on the number of confirmed cases in each city in the first half of 2020, and take the first half of 2020 as the time when the shock occurred. The data on the cumulative number of confirmed cases in each city is from the COVID-19 Timely Dynamic Tracking Database of SINA.

**Table A3.** Descriptive statistics for low-income families and families without Internet

|  | | *Low-income family* | | Total |
| --- | --- | --- | --- | --- |
|  |  | No (=0) | Yes (=1) |  |
| *Internet* | No (=0) | 1039 | 671 | 1710 |
|  | Yes (=1) | 9040 | 1346 | 10386 |
| Total | | 10079 | 2017 | 12096 |

Notes: *Low-income family* is a dummy variable, if the value is 1, it means that the family is relatively poor or very poor in the self-assessment. *Internet* equals 1, which means there is a computer at home with Internet access, otherwise it is equal to 0. The numbers in the table indicate the number of observations in different groups.

**Table A4.** Summary statistics of added control variables in robustness checks

| Variable | Definition | N | Mean | S.D. |
| --- | --- | --- | --- | --- |
| Student-parent relationship | Relationship between students and parents (= 1 good; = 0 not good) | 12096 | 0.851 | 0.356 |
| parental education expectations | =1 Expect the child to receive a master's or doctoral education; =0 otherwise | 12096 | 0.485 | 0.500 |
| Father's attitude | Whether the father is strict with his children (=1 yes; =0 no) | 12096 | 0.136 | 0.343 |
| Mother's attitude | Whether the mother is strict with her children (=1 yes; =0 no) | 12096 | 0.115 | 0.319 |
| Extracurricular activities | Whether the student participates in extracurricular activities or club activities at school (=1 yes; =0 no) | 12096 | 0.275 | 0.446 |
| Award | Whether the student has received an award for the school's outstanding student (=1 yes; =0 no) | 12096 | 0.456 | 0.498 |
